# Aneurysm of the inferior vena cava with thrombosis

**DOI:** 10.1002/ccr3.1321

**Published:** 2018-01-13

**Authors:** Masashi Inoue, Takeshi Sudo, Megumi Yamaguchi, Shingo Seo, Tatsuya Miyamoto, Toshihiro Misumi, Wataru Shimizu, Toshimitsu Irei, Takahisa Suzuki, Takashi Onoe, Yosuke Shimizu, Takao Hinoi, Hirotaka Tashiro

**Affiliations:** ^1^ Department of Surgery National Hospital Organization Kure Medical Center Chugoku Cancer Center Kure Japan

**Keywords:** Inferior vena cava (IVC) aneurysms, IVC filter, lumber vein, resection, retroperitoneal hematoma, thrombosis

## Abstract

Inferior vena cava (IVC) aneurysms are extremely rare. Patients can be asymptomatic, have thrombosis, rupture, or pulmonary embolism. Thrombosis of the IVC aneurysm may mimic a retroperitoneal tumor. Surgical treatment of abdominal venous aneurysms with thrombosis is warranted and is necessary for the management of intraoperative bleeding and thrombosis.

## Introduction

Inferior vena cava (IVC) aneurysms are extremely rare. IVC aneurysms can present with significant clinical complications such as thrombosis, pulmonary embolism, and bleeding 1, 2, 3. These various entities may be difficult to differentiate clinically, and the literature offers no clear guidance as to the selection of conservative or surgical treatment.

We report a case of a saccular IVC aneurysm with thrombosis that mimicked a retroperitoneal hematoma, which was believed to originate from the IVC.

## Case Report

A 56‐year‐old female presented to our hospital with a right abdominal palpable tumor and pain of 7 days’ duration. She had slight tenderness at the right abdomen on physical examination. Laboratory tests that included a coagulation test showed no remarkable findings. Serum levels of carcinoembryonic antigen and carbohydrate antigen 19‐9 were 0.9 ng/mL and 14 U/mL, respectively.

**Figure 1:**
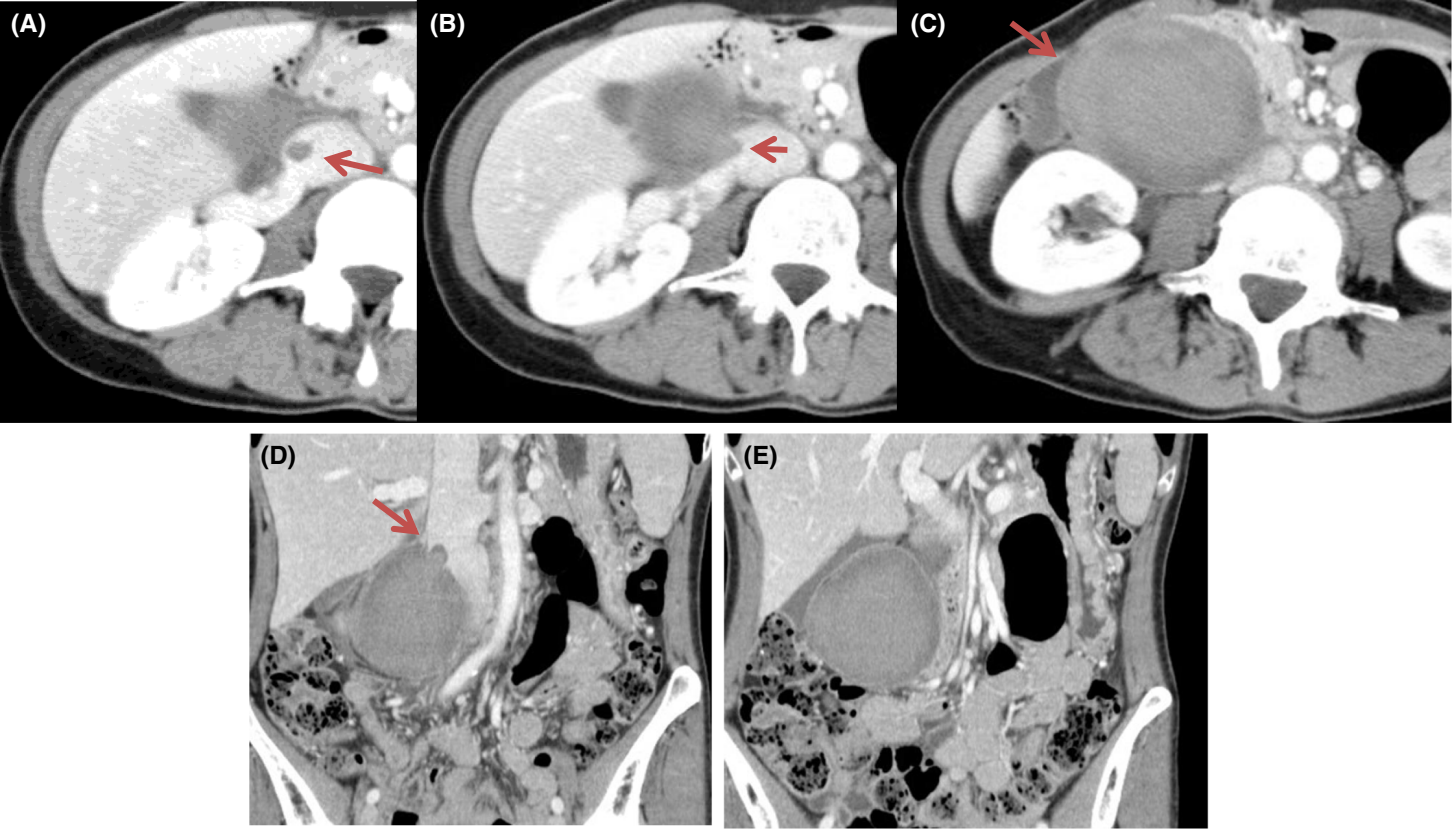
(A–C) Contrast‐enhanced axial CT image demonstrating a 73 × 63 mm homogenous mass lateral to the duodenal second portion and medial to the right kidney with IVC thrombosis. (D, E) Contrast‐enhanced coronal CT image.

**Figure 2:**
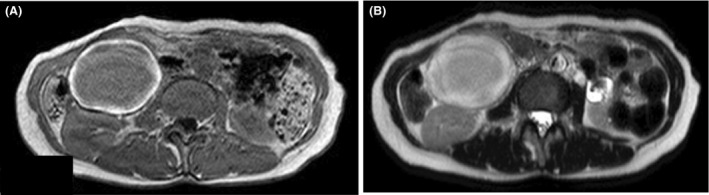
Magnetic resonance (MR) imaging revealed a mass with ring‐like enhancement on (A) T1‐ and (B) T2‐weighted images, and thrombus in the IVC.

**Figure 3:**
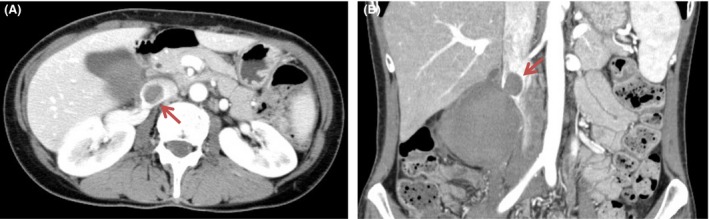
After 4 days, Enhanced CT revealed the proliferation of the thrombus, although there was no remarkable change in the mass and there was proliferation of the thrombus.

Contrast‐enhanced computed tomography (CT) revealed a 73 × 63 mm homogenous mass lateral to the second part of the duodenum and medial to the right kidney with IVC thrombosis (Fig. 1). Anticoagulant therapy was performed with heparin, which was infused at 5000 U/day for first 24 h. The dosage was then increased to 20,000 U/day until the APTT value increased to twice the standard value. Magnetic resonance (MR) imaging revealed a mass with ring‐like enhancement on T1‐ and T2‐weighted images, and thrombus was seen in IVC (Fig. 2). After 4 days, enhanced CT was used to evaluate the mass and thrombus. It showed that there was no remarkable change in the mass, but there was proliferation of the thrombus (Fig. 3).

Therefore, based on the diagnosis of retroperitoneal tumor with thrombosis of the IVC, an IVC filter was installed, followed by resection of the tumor and thrombectomy.

Intraoperatively, the hematoma was seen below the liver between the second part of the duodenum and the right kidney. The tumor was separated on all sides while carefully avoiding damage to the right renal artery and other veins. The communication between the hematoma and IVC was identified. The right and left veins, and VC were clamped above and below the tumor before the tumor was excised, and the thrombus was seen extruding from the neck of the tumor. After thrombectomy, the venotomy was sutured with fine nonabsorbable sutures. During venotomy, about 1000 mL of rapid blood loss occurred, although several main veins were clamped. This bleeding was assumed to be from the lumbar veins.

**Figure 4:**
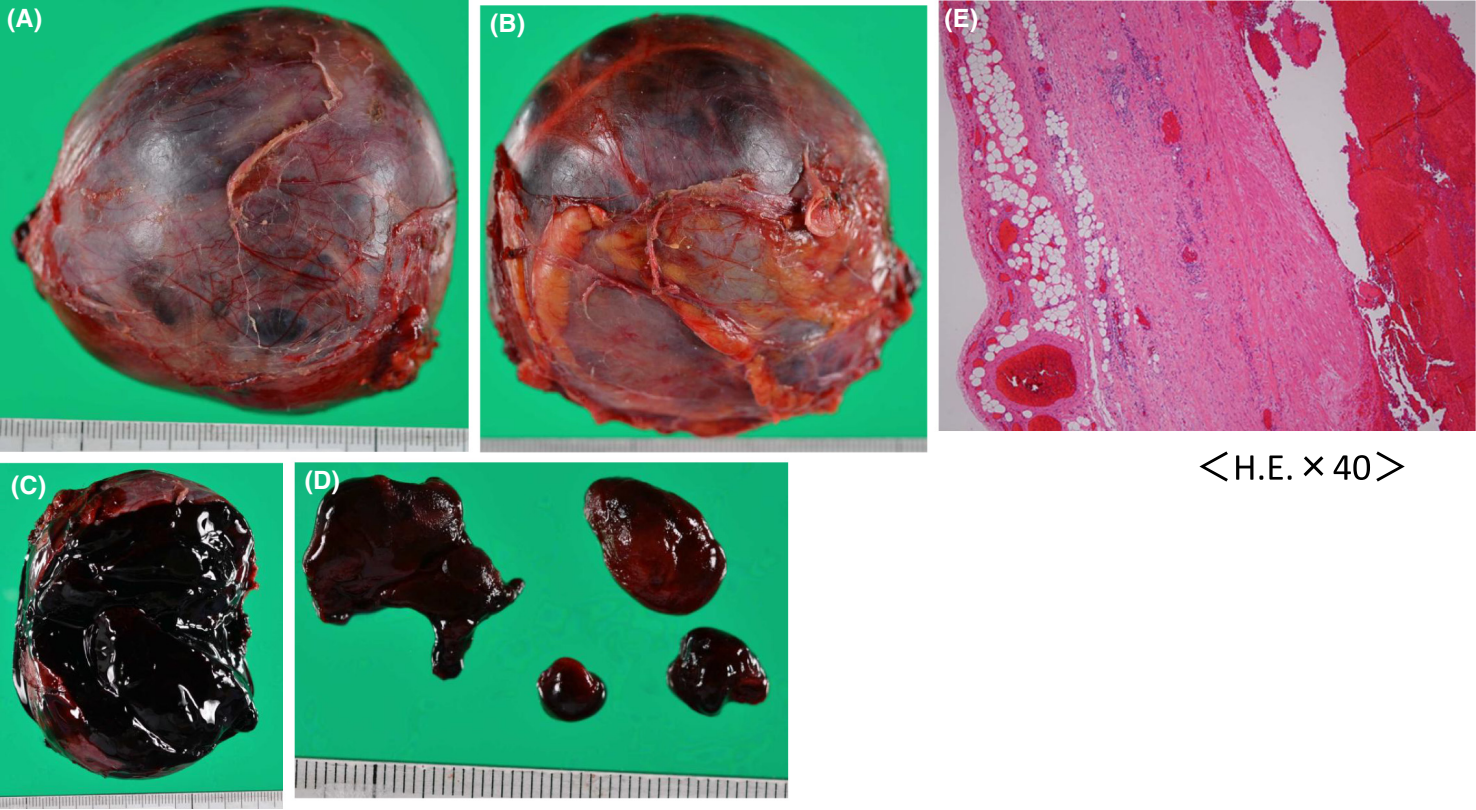
(A–C) Tumor containing hematoma; (D) Thrombosis; (E) Histopathological examination found smooth muscle cells in layers of the tumor wall and hematoma. Therefore, this tumor was diagnosed as an aneurysm of the IVC with thrombosis.

Histopathological examination found smooth muscle cells in layers of the tumor wall and hematoma. Therefore, this tumor was diagnosed as aneurysm of the IVC with thrombosis (Fig. 4).

**Figure 5:**
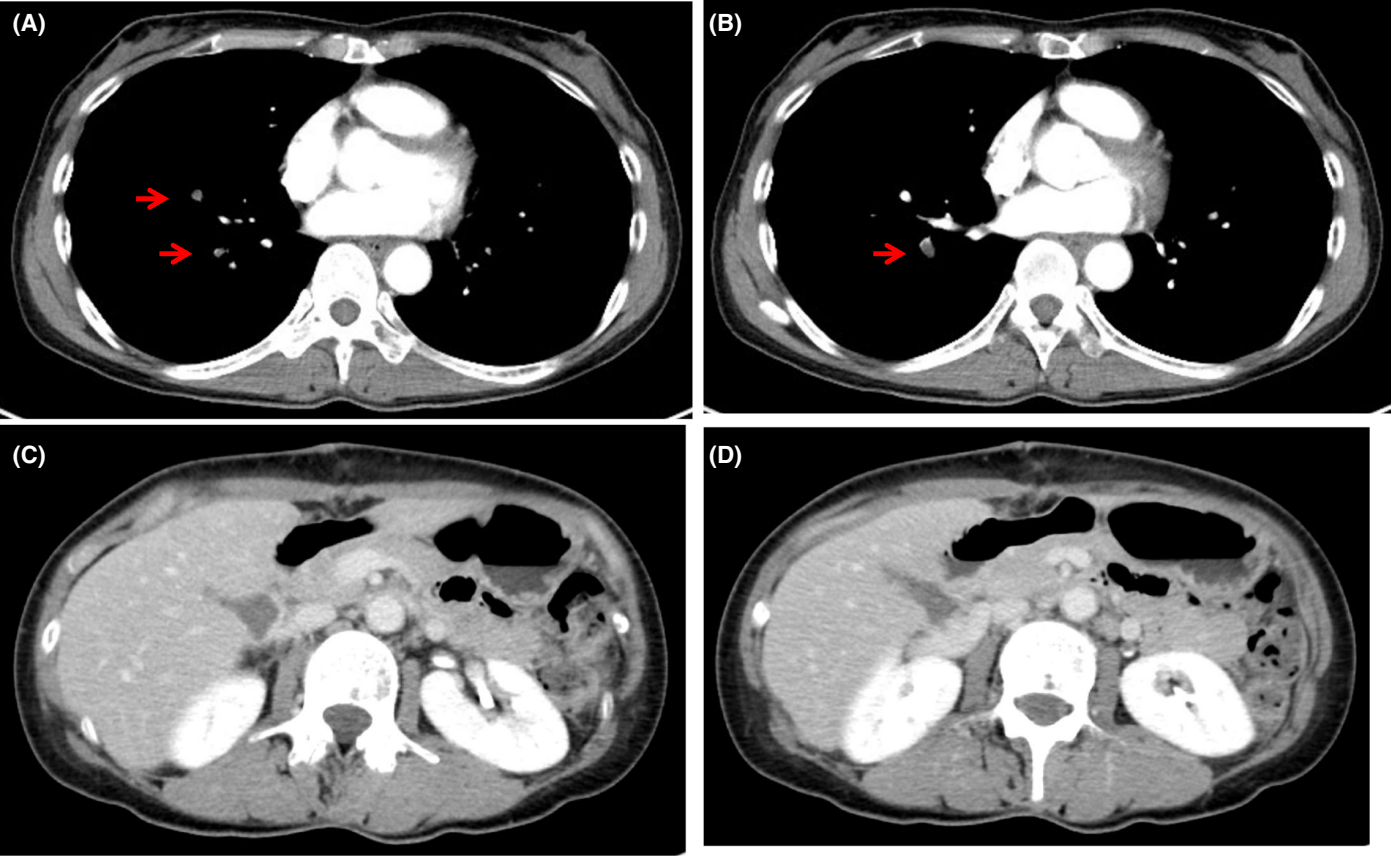
(A, B) On day 7 after the operation, CT revealed thrombosis of the peripheral pulmonary artery(A,B) but did not sow IVC thrombosis(C,D).

On day 4 after the operation, the IVC filter was retrieved. On day 7 after the operation, CT revealed thrombosis of the peripheral pulmonary artery, although there was no thrombosis of the reconstructed caval segment (Fig. 5). The patient was started on anticoagulation therapy and continued to be asymptomatic. Three months later, CT did not detect thrombosis, and anticoagulation therapy was stopped. At the present time, one year after the operation, the patient is asymptomatic without any sign of recurrence.

## Discussion

Venous aneurysms are rare and defined as persistent isolated venous dilatation that is twice the normal diameter of 1.5–3.7 cm 4, 5, 6. The pathological factors that cause IVC aneurysm include trauma, inflammatory processes, long‐standing hypertension, and congenital defects 7. Various imaging modalities have been used for the diagnosis of IVC aneurysm. However, when the lumen of the IVC aneurysm is completely obstructed by thrombosis, the diagnostic value of these imaging studies is significantly impaired. Such venous anomalies could be confused with retroperitoneal tumors, such as sarcomas, enlarged lymph nodes, neurogenic tumors and renal carcinoma, and primary IVC tumors, including leiomyosarcoma and leiomyoma 8.

The signal within the lesion on MRI can vary over time, indicating time‐related changes in hemoglobin levels 9. High signal intensity on T1‐weighted images is attributable to the presence of methemoglobin within the hematoma. In our case, the hematoma was assumed to have developed over 7–30 days. However, MRI could not discriminate between a benign and malignant retroperitoneal tumor. Positron emission tomography may be useful and ultrasound or CT‐guided fine‐needle aspiration may demonstrate the malignant nature of a tumor 10, 11. Surgical intervention allows for complete removal of the sac with primary venous closure and provides tissue for an accurate histological diagnosis 12.

According to a recent literature review by Montero‐Baker et al. in 2014, 53 total cases of IVC aneurysm have been reported 13. In this review, four types of IVC aneurysms were described, according to the Gradman and Steinberg classification of the IVC 2; surgical consideration should be given based on high rates of thromboembolic complications and a non‐negligible risk of rupture. Surgical treatment of IVC aneurysms has generally consisted of complete or partial excision and repair of the IVC defect. There are various treatment options depending on the site and the presentation vessel, and type of aneurysm. All alternatives must be utilized to provide continuity of the vascular structure to protect normal blood flow. Primary repair, graft interposition 3, patch plasty 14, or endovascular interventions are the treatment options for IVC aneurysms.

With so few reports on IVC aneurysms, the literature offers no clear risk for a surgical procedure. In the case of concomitant massive thrombosis of the proximal veins, placement of a suprarenal IVC filter should be considered. In our case, postoperative pulmonary embolism occurred after thrombectomy without thrombosis of the reconstructed caval segment. We suspect that the IVC filter trapped these thrombi liberated by the operation and prevented fatal pulmonary thromboembolism. In the case of a huge hematoma, all lumbar vein ligation procedures might be impossible. In our case, during resection of the aneurysm, massive bleeding from the lumbar vein had to be controlled by finger compression. This procedure controlled the bleeding and allowed time for suturing. Because of the nature of a small‐necked tumor, another procedure such as side clamping can also be used to stop the bleeding 15.

In conclusion, the diagnosis of IVC aneurysm should be considered in the differential diagnosis of retroperitoneal tumors, when the IVC lumen is completely obstructed by thrombosis. During the perioperative period of surgery for abdominal venous aneurysms with thrombosis, an IVC filter is useful to prevent pulmonary thromboembolism, and the most suitable surgical technique to reduce lumber vein bleeding must be chosen.

## Consent

Written informed consent was obtained from the patient for publication of this case report and any accompanying images. A copy of the written consent is available for review by the editor in chief of this journal.

## Conflict of Interest

There is no potential conflict of interests to disclose.

## Authorship

MI: wrote the manuscript. MI and TS: designed the study. MY, SS, TM, WS, TI, TS, TO, YS, TH, HT: proofread the manuscript.
